# Applying Rank Sum Ratio (RSR) to the Evaluation of Feeding Practices Behaviors, and Its Associations with Infant Health Risk in Rural Lhasa, Tibet

**DOI:** 10.3390/ijerph121214976

**Published:** 2015-12-01

**Authors:** Zhenjie Wang, Shaonong Dang, Yuan Xing, Qiang Li, Hong Yan

**Affiliations:** 1 Institute of Population Research/WHO Collaborating Center on Reproductive Health and Population Science, Peking University, Beijing 100871, China; zhenjie.wang@pku.edu.cn; 2 Department of Epidemiology and Health Statistics, School of Medicine, Xi’an Jiaotong University, Xi’an 710061, China; tjlq@mail.xjtu.edu.cn (Q.L.); yanhonge@mail.xjtu.edu.cn (H.Y.); 3 Xi’an Center for Disease Control and Prevention, Xi’an 710061, China; tjxxyy@163.com

**Keywords:** rank sum ratio, feeding practices index, infant health, Tibet

## Abstract

To evaluate the status of feeding practices and analyze the association between feeding practice and health status among Tibetan infants, a cross-sectional survey of 386 women with children aged under 24 months was conducted in rural areas surrounding Lhasa, Tibet. All participants were selected using simple random sampling and were interviewed face-to-face by trained interviewers. Mothers were interviewed to collect information on their feeding practices. A feeding practices index was created using the rank sum ratio method. Most of the infants had been or were being breastfed at the time of the interview. The feeding practices index was significantly and inversely associated with the prevalence of acute upper respiratory infection, and the odds ratio for the qualified feeding practices index *vs.* the non-qualified feeding practices index was 0.43 (95% confidence interval: 0.20–0.94). There were no measurable associations observed between acute upper respiratory infection, diarrhea, and the feeding practices index after controlling for selected factors. The method of rank sum ratio provides a flexible way to evaluate feeding practices and is easy to understand. Furthermore, appropriate infant feeding practices might play a protective role in Tibetan infants’ health.

## 1. Introduction

Breastfeeding and complementary feeding practices are fundamental to a child’s nutrition, survival, and developmental status [1,2]. Every year, as many as 55% of the deaths of children under five years of age caused by diarrheal disease and acute respiratory infections may be the result of malnutrition. However, fewer than 35% of infants worldwide are exclusively breastfed for even the first four months of life, and complementary feeding practices are frequently ill-timed, inappropriate, and unsafe [3]. Furthermore, an association between the quality of feeding practices of infants and nutritional status is difficult to establish. Depending on the type of complementary feeding and overall living conditions, the effect of maternal feeding factors on children’s nutritional status can vary considerably [4]. In addition, feeding practices are often complex, changing with a child’s age [5]. Ruel and Menon proposed an Infant and Child Feeding Index (ICFI) based on an age-specific scoring system that gives points for positive practices in terms of breastfeeding, bottle feeding, meal frequency, and food diversification for 6–36 months [6,7,8,9]. This index has been used to assess the effect of complementary feeding practices on child growth [8,9,10]. Although the ICFI is a reasonable method for evaluating infant feeding practices, it excludes infants under six months of age and follows a strict study design to collect data on infant feeding practices.

Since research design limitations prevent the ICFI from being used to evaluate feeding practices for children aged under six months, the present study used data from a cross-sectional study conducted in rural areas of Lhasa, Tibet, to assess a feeding practices index using the rank sum ratio (RSR) method and to analyze the association between feeding practices and child health status.

## 2. Methods

### 2.1. Data and Survey Design

Data were derived from a cross-sectional survey designed to document the health status of children aged under 24 months and their mothers’ dietary and nutrient intake. We calculated the sample size based on the rates of diarrhea (67.2%) and upper respiratory infection (67.3%) reported in the 2002 NNHS and the rate of breastfeeding (85%) in the Development of Chinese Children (2001–2010). The sample size equations were as follows: α = 0.05, δ = |p − π| = 0.05, followed by n=μα22π(1−π)δ2. The required sample sizes were 339, 338, and 196, respectively. We chose the maximum sample size (339), and expected a 20% no-response rate. The final sample size was 406. Details of the study design and conduct are described elsewhere [11]. The survey samples were drawn using simple random sampling based on a list of children aged under 24 months and their mothers from local health care system in rural areas surrounding Lhasa. Lhasa is the capital of Tibet, which is located on the Qinghai-Tibet Plateau, with a mean altitude of 3685 m above sea level. The participants for maternal and child healthcare were children less than 24 months old and their mothers. Participants were selected using simple random sampling, and the mothers were interviewed face-to-face by trained professional interviewers from May to August 2008. Complete data on a total of 386 eligible children and their mothers (95.1%) were obtained from the survey. The major reasons for those who did not finish this survey were: (1) they refused to attend this survey; and (2) mothers were still working outside the survey areas during the survey time.

### 2.2. Ethics

The study protocol was approved by the Ethics Review Committee at Xi’an Jiaotong University College of Medicine (Approval No. 2007015).

### 2.3. Feeding Practices Index

In this survey, we collected data on children and their mothers, including child healthcare, feeding information, the behavior and attitude of the mothers towards feeding, and mothers’ healthcare. Moreover, sociodemographic information, including parental age, years of education, maternal occupation, and family size, was collected by a self-designed questionnaire described in previous studies [12,13]. In the present analysis, we primarily used the feeding data, which covered breastfeeding practice and complementary feeding practices including the time, frequency and type of food introduced. All feeding information was obtained by interviewing the mothers. Participants were re-interviewed when feeding practices information was missing from the questionnaire.

Former applications of the ICFI have usually comprised breastfeeding, bottle feeding, dietary diversity (past 24 h), food group frequency (past seven days), and feeding frequency [7,8,9]. Since the information we collected did not support the calculation of the ICFI as it was performed previous studies, in the present study, we used an index including current feeding practice and complementary feeding practices, and based recommendations for feeding children on a flexible statistical method, RSR. The feeding practices index included variables for current feeding practice and complementary feeding practice that were sorted according to World Health Organization recommendations for infant feeding practices. In the present research, the feeding practices index was evaluated by using RSR [14]. RSR is a comprehensive evaluation method developed by Tian Fengdiao. The validation and rationality of RSR method has been proved by Tian Fengdiao [14]. The fundamental theory behind the method is that a dimensionless statistical indicator (RSR) is calculated from an *n* × *m* matrix using rank conversion. After this calculation, the distribution of RSR could be explored using parametric statistical methods. Generally, the RSR indicator ranges from zero (worst) to one (best) and follows a normal distribution. Additionally, subjects’ status (worst/best) could be evaluated using the RSR order or a set of ordinal classification. Classifications of RSR values use empirical percentiles based on the standard normal deviation, as suggested by Tian Fengdiao (Table 1) [14]. Strengths of RSR include the following: (a) it is easy to use, and no additional data are required; (b) it is flexible and can be combined with other statistical methods; and (c) it is useful for comparing differences or finding associations. A limitation of RSR is that it requires that there be no missing values in the *n* × *m* matrix.

**Table 1:** Percentile of Rank Sum Ratio (RSR) classifications ^a^.

Number of Classification	Percentile of RSR						
3	<15.866	15.866~	84.134~				
4	<6.681	6.681~	50~	93.319			
5	<3.593	3.593~	27.425~	72.575~	96.407~		
6	<2.275	2.275~	15.866~	50~	84.134~	97.725~	
7	<1.168	1.618~	10.027~	33.360~	67.003~	89.973~	98.352~

^**a**^ Percentile of Rank Sum Ratio (RSR) suggested by Tian Fengdiao [14].

In the current study, variables and values used to create the feeding practices index by the infant’s age are presented in Table 2. The infant feeding index was calculated as follows basing on variables in Table 2: First, an *m* × *n* matrix was created: n means the number of variables, m means the number of infant subjects. Second, rank conversion was performed: in the current study, all variables were valued as high-quality variables, meaning that the lowest value indicates the worst outcome. Therefore, the variables were sorted in ascending order in the rank conversion process. Third, the ranks (*R_ij_*) were summed over rows, giving each column or variable a score, and then summed over these columns.

(1) $${RSR}_{i}=\frac{\sum_{j=1}^{m}R_{ij}}{m\times n}\left(i=1\cdots n\right),\;RSR=\sum_{i=1}^{n}{RSR}_{i}$$

Furthermore, every variable individually used in the feeding practices index was weighted by the independent weight method in each age group [15]. A weighted RSR was then calculated by

(2) $${RSR}_{w}=\frac{\sum WR}{n\sum W}$$

Fourth, we explored the distribution of the infant feeding index (Shapiro–Wilk = 0.994, *p* = 0.20).

### 2.4. Infant Health Status

Infant health status information, including acute upper respiratory infection and diarrhea, was collected as part of the survey. Two-week prevalence of acute upper respiratory infection or diarrhea was calculated based on whether these diseases occurred during the two weeks preceding the interview. Overall prevalence of these diseases was calculated based on whether these diseases occurred at any point before the interview.

**Table 2:** Variables and value used to create the feeding practices index by the Rank Sum Ratio (RSR) according to infant’s age distribution ^a^.

Variables	0–5.99 Months	6–8.99 Months	9–11.99 Months	≥12 Months
Breastfeeding	No = 0; Yes = 1	No = 0; Yes = 1	No = 0; Yes = 1	No = 0; Yes = 1
Still breastfeeding	No = 0; Yes = 1	No = 0; Yes = 1	No = 0; Yes = 1	No = 0; Yes = 1
Beginning breastfeeding (h)	time: 0 → Max	time: 0 → Max	time: 0 → Max	time: 0 → Max
	value: 1 → 0	value: 1 → 0	value: 1 → 0	value: 1 → 0
Breast milk is enough or not (in 4 months after birth )	No = 0; Yes = 1	Early introduction = 0; Yes = 1	Early introduction = 0; Late introduction = 0; Recommended introduction = 1	Early introduction = 0; Late introduction = 0; Recommended introduction = 1
Introduced complementary food ^b^	No = 1; Early introduction = 0	Early introduction = 0; Yes = 1	Early introduction = 0;	Early introduction = 0;
			Late introduction = 0;	Late introduction = 0;
			Recommended introduction = 1	Recommended introduction = 1
Frequency of added complementary food ^c^	No = 6; ≤1 time/month = 5;	≤1 time/month = 0;	≤1 time/month = 0;	≤1 time/month = 0;
	2–3 times/month = 4;	2–3 times/month = 1;	2–3 times/month = 1;	2–3 times/month = 1;
	>3 times/month = 3;	>3 times/month = 2;	>3 times/month = 2;	>3 times/month = 2;
	≤1 time/week = 2;	≤1 time/week = 3;	≤1 time/week = 3;	≤1 time/week = 3;
	2–3 times/week = 1;	2–3 times/week = 4;	2–3 times/week = 4;	2–3 times/week = 4;
	>3 times/week = 0;	>3 times/week = 5;	>3 times/week = 5;	>3 times/week = 5;

^**a**^ The higher value means the better rank for RSR; ^**b**^ Included sweet water, Tibetan milk tea, Tibetan salt cream tea, Zanba, porridge, egg, fresh milk, milk powder, bean products, fish, any kinds of meat (beef, mutton, pork, and chicken), fresh vegetables and fresh fruits; ^**c**^ Included egg, and any kinds of meat (beef, mutton, pork, and chicken); based on recommended introduced month of complementary food, we valued with different values.

### 2.5. Quality Control

The interviewers (physicians from the Department of Public Health, School of Medicine, Tibet University) were trained to standardize the administration of the questionnaire and the recording of anthropometric measurements. The interviewers were trained in the field for at least one week prior to administering the survey. A pilot survey performed before the formal survey included all of the formal survey items relevant for the present analysis. Two investigation teams were established—each consisting of four members and a supervisor. At least two members of each team were Tibetan and were able to communicate in both Tibetan and Chinese. During the survey, a data checking system was employed, through which all interviewers checked their own data and cross-checked each other’s data, and supervisors subsequently checked all data. Participants were re-interviewed when inconsistent answers to key questions or missing values were identified in this process. Information on the exact age of the child was based on permanent residence registration and/or immunization records, which contain birth dates.

### 2.6. Statistical Analysis

In this study, we first grouped the participants based on the National Nutrition and Health Investigation’s criteria for nutrition and health status among Chinese children aged 0–6 years [16]. Participants were divided into two categories (not qualified feeding practices: <60th percentile; qualified feeding practices: ≥60th percentile). We then further divided the subjects into three subcategories (poor feeding practices: <percentile 15.866; medium feeding practices: >percentile 15.866 and <percentile 84.134; good feeding practices: ≥percentile 84.134), as suggested by Tian Fengdiao [14]. Finally, the subjects were divided into tertile/quartile categories according to the continuous feeding practices index to investigate whether the association between the feeding index and health status was stable. Differences in the prevalence of diseases between those categorized as having qualified feeding practices and those with non-qualified feeding practices were examined using the chi-square test. Trends in the prevalence of diseases according to the three subcategories (poor, medium, and good) of the feeding practices index were examined by the Cochran–Armitage trend test. Logistic regression analysis was used to estimate the odds ratios (ORs) and 95% confidence intervals (CI) of acute upper respiratory infection and diarrhea for each category, with the lowest category as the reference group. The 95% CI was calculated using the standard error of the logistic regression coefficient. Based on a previous report [17], statistical adjustments were made for infant’s age (0–5.99, 6–8.99, 9–11.99, and ≥12 months), infant’s sex, maternal years of education (<1, 1–8 and ≥9), maternal occupation (farming and animal husbandry only or other), paternal years of education (<1, 1–8 and ≥9), and paternal occupation (farming and animal husbandry only or other). Trends in the association between diseases and the feeding practices index were assessed with ordinal scores assigned to the feeding practices index categories. Results were considered statistically significant if the two-sided *p*-value was <0.05. Statistical analyses were performed using SAS version 9.2 (SAS Institute Inc., Cary, NC, USA).

## 3. Results

All 386 infants were native to and lived in Tibet. Most of the infants were the first children in their families, had been introduced to complementary food, and had been or were being breastfed at the time of the interview. Their families were usually large and made a living by farming or raising animals, as well as other means (Table 3).

**Table 3:** Characteristics of selected subjects.

Characteristic	*n* (%)
Infant age, months	
0–5.99	60 (15.5)
6–8.99	46 (11.9)
9–11.99	39 (10.1)
≥12	241 (62.4)
Gender	
Boys	196 (50.8)
Girls	190 (49.2)
Rank of infant in each household	
1	225 (58.3)
2	140 (36.3)
≥3	21 (5.4)
Infants’ feeding pattern for 4 months	
Almost exclusive breastfeeding	3 (0.8)
Partial breastfeeding	339 (87.8)
Weaning	44 (11.4)
Infant feeding index	
Dichotomy	
No qualified	323 (83.7)
Qualified	63 (16.3)
Tripartite	
Poor	60 (15.5)
Medium	263 (68.1)
Good	63 (16.3)
Maternal educational years	
<1	122 (31.6)
1–8	183 (47.4)
≥9	81 (21.0)
Maternal occupation	
Farming and animal husbandry only	277 (72.1)
Others	109 (28.2)
Paternal educational years	
<1	92 (23.8)
1–8	198 (51.3)
≥9	96 (24.9)
Paternal occupation	
Farming and animal husbandry only	225 (58.3)
Others	161 (41.7)
Family size	Median (25th%–75th%)
	5 (4–7)

The two categories of the feeding practices index (non-qualified, and qualified) showed a strongly significant inverse association with the prevalence of acute upper respiratory infection, without adjustment. Although this strong inverse association was attenuated after adjustments, it remained significant (*p* = 0.03). The risk of having an acute upper respiratory infection tended to be reduced in children who had a qualified feeding practice, compared with children without a qualified feeding practice (ORs = 0.43, 95% CI: 0.20–0.94). In contrast, a significant inverse association was found with the prevalence of diarrhea before controlling for selected factors, but this association disappeared after adjustment (Table 4). A similar phenomenon was observed in the association between the three categories of the feeding practices index (poor, medium, and good feeding practices) and the prevalence of acute upper respiratory infection. Before adjustment, there was a significant inverse association; however, this association disappeared after controlling for selected factors. The three categories of the feeding practices index were not measurably associated with the two-week prevalence of acute upper respiratory infection or diarrhea (Table 5). When the participants were divided into tertile/quartile categories according to the continuous feeding practices index, the associations between infant feeding practices and health status remained consistent with the above results.

**Table 4:** Disease status as related to two categories of feeding practices index.

	Categories of Infant Feeding Index		*p*
	Not Qualified	Qualified	
Acute upper respiratory infection			
Two week prevalence (%)	26.6	20.6	0.32 ^a^
Unadjusted OR (95% CI)	1.00 (reference)	0.72 (0.37–1.38)	0.20
Adjusted OR (95% CI) ^b^	1.00 (reference)	0.56 (0.23–1.36)	0.20
Prevalence (%)	70.6	36.5	<0.01 ^a^
Unadjusted OR (95% CI)	1.00 (reference)	0.24 (0.14–0.42)	<0.001
Adjusted OR (95% CI) ^b^	1.00 (reference)	0.43 (0.20–0.94)	0.03
Diarrhea			
Two week prevalence (%)	18.0	22.2	0.43 ^a^
Unadjusted OR (95% CI)	1.00 (reference)	1.31 (0.68–2.52)	0.43
Adjusted OR (95% CI) ^b^	1.00 (reference)	1.24 (0.49–3.15)	0.65
Prevalence (%)	62.9	41.3	0.001 ^a^
Unadjusted OR (95% CI)	1.00 (reference)	0.42 (0.24–0.72)	0.002
Adjusted OR (95% CI) ^b^	1.00 (reference)	0.73 (0.34–1.56)	0.42

^**a**^ Chi-Square test; ^**b**^ adjusted for infant age, gender of infant, maternal educational years, maternal occupation, paternal educational years and paternal occupation.

**Table 5:** Disease status as related to three categories of feeding practices index.

	Categories of Infant Feeding Index			*p_trend_*
	Poor	Medium	Good	
Acute upper respiratory infection				
Two weeks prevalence (%)	31.7	25.5	20.6	0.16 ^**a**^
Unadjusted OR (95% CI)	1.00 (reference)	0.74 (0.40–1.36)	0.56 (0.25–1.28)	0.16
Adjusted OR (95% CI) ^**b**^	1.00 (reference)	0.78 (0.42–1.47)	0.46 (0.16–1.28)	0.16
Prevalence (%)	65.0	71.9	36.5	0.0007 ^**a**^
Unadjusted OR (95% CI)	1.00 (reference)	1.38 (0.76–2.49)	0.31 (0.15–0.65)	<0.001
Adjusted OR (95% CI) ^**b**^	1.00 (reference)	1.45 (0.79–2.67)	0.60 (0.24–1.53)	0.57
Diarrhea				
Two weeks prevalence (%)	13.3	19.0	22.2	0.21 ^**a**^
Unadjusted OR (95% CI)	1.00 (reference)	1.53 (0.68–3.42)	1.86 (0.72–4.81)	0.21
Adjusted OR (95% CI) ^**b**^	1.00 (reference)	1.49 (0.66–3.38)	1.77 (0.54–5.81)	0.31
Prevalence (%)	55.0	64.6	36.7	0.11 ^**a**^
Unadjusted OR (95% CI)	1.00 (reference)	1.50 (0.85–2.64)	0.56 (0.28–1.18)	0.11
Adjusted OR (95% CI) ^**b**^	1.00 (reference)	1.42 (0.79–2.55)	1.00 (0.40–2.48)	0.73

^**a**^ Cochran–Armitage trend test; ^**b**^ adjusted for infant age, gender of infant, maternal educational years, maternal occupation, paternal educational years and paternal occupation.

## 4. Discussion

To the best of our knowledge, little is known regarding an association between a feeding practices index created using the RSR method and healthy status among Tibetan infants aged 0–24 months. Our study introduced a new and reasonable method to create a feeding practices index and provides basic data on the practices of Tibetan infants. Like other infant feeding indices [6,7,8,9], our feeding practices index included basic feeding practices, such as breastfeeding, and the frequency of intake of complementary foods. Our index differed from the ICFI in that the ICFI was created without considering inter-association within feeding behaviors but our index did consider this. Additionally, our scoring system expanded the infant age group covered to include those aged 0–24 months, which was wider than the ICFI’s age range and provided much more valuable information. Therefore, this evaluation method provided a reasonable pathway to assess feeding practices for Tibetan infants aged 0–24 months, and it also provided a flexible method of evaluation without restriction of the study’s design.

We also evaluated the association between our infant feeding index and infant health status. Our scoring system was found to have strong positive associations with the recommended introduction of complementary foods, suggesting that our feeding practices index could indicate the circumstances of a child’s feeding. The ICFI has been used in Latin American countries [6] and rural China [10] to assess the effects of feeding practices on child growth. However, in these previous studies [6,7,8,9], the researchers did not address the association between the ICFI and children’s health status. A previous epidemiological study suggested that exclusive breastfeeding protects against rotavirus diarrhea in infants [18], but findings on this topic are inconsistent [19]. Although we did not analyze the association between breastfeeding practices and diarrhea, we found that there was a significant decreasing trend in the prevalence of diarrhea as the feeding practices index increased, but this trend disappeared following adjustments. We also found a measureable negative association between acute upper respiratory infection and the feeding practices index. These results suggest that feeding practice might be associated with the prevalence of acute upper respiratory infection, and most likely with diarrhea, indicating a relationship between feeding practice and health outcomes of children. We speculate that nutritionally inadequate feeding practices might be responsible for poor Tibetan infant health.

There are some limitations in the present study. First, although the cross-sectional design of the survey yields information about the nutritional status of Tibetan mothers with children under two years of age, this design could not provide direct evidence of causality [20]. Therefore, this characteristic of the study design should be noted when the results are used in other research. Second, the RSR method required that there be no missing values on the variables used for evaluation. Finally, unfortunately, we did not collect data on children’s nutritional status, such as weight and length, because of limitations of the study design. This meant that we were not able to investigate the relationship between RSR and children’s nutritional status. However, this method was useful and flexible without imposing restrictions on the collected items. Although there are some limitations in the present study, it nevertheless provides valuable information on feeding practice and its associations with Tibetan infant health.

## 5. Conclusions

The Rank Sum Ratio (RSR) is a useful and flexible method on evaluating Tibetan infant feeding practices. Appropriate infant feeding plays a protective role in acute upper respiratory and diarrhea among Tibetan infants aged 0–24 months.
